# Synovial Predictors of Differentiation to Definite Arthritis in Patients With Seronegative Undifferentiated Peripheral Inflammatory Arthritis: microRNA Signature, Histological, and Ultrasound Features

**DOI:** 10.3389/fmed.2018.00186

**Published:** 2018-07-03

**Authors:** Stefano Alivernini, Barbara Tolusso, Luca Petricca, Laura Bui, Clara Di Mario, Maria R. Gigante, Gabriele Di Sante, Roberta Benvenuto, Anna L. Fedele, Francesco Federico, Gianfranco Ferraccioli, Elisa Gremese

**Affiliations:** ^1^Division of Rheumatology, Fondazione Policlinico Universitario A. Gemelli, IRCCS, Catholic University of the Sacred Heart, Rome, Italy; ^2^Institute of Pathology, Fondazione Policlinico Universitario A. Gemelli, IRCCS, Catholic University of the Sacred Heart, Rome, Italy

**Keywords:** undifferentiated peripheral inflammatory arthritis, synovial tissue biopsy, ultrasonography, microRNA, predictor

## Abstract

**Objectives:** To examine synovial tissue (ST) predictors of clinical differentiation in patients with seronegative undifferentiated peripheral inflammatory arthritis (UPIA).

**Methods:** Fourty-two patients with IgA/IgM-Rheumatoid Factor and anti-citrullinated peptide antibodies negative UPIA, naive to Disease-Modifying Anti-Rheumatic Drugs, underwent Gray Scale (GSUS) and power Doppler (PDUS) evaluation and Ultrasound (US) guided ST biopsy. CD68, CD3, CD21, CD20, and CD31 synovial expression was evaluated by immunohistochemistry. Whole ST microRNA expression was assessed using miScript miRNA PCR Array. Peripheral blood (PB) and synovial fluid (SF) IL-6, VEGF-A, and VEGF-D levels were measured by ELISA and ST TNF expression was assessed by RT-PCR. Each patient was prospectively monitored and classified at baseline and within 1 year as UPIA, Rheumatoid Arthritis (RA), Spondyloarthritis (SpA) or Psoriatic Arthritis (PsA), respectively.

**Results:** At baseline, CD68^+^ cells were the most common cells within the lining layer (*p* < 0.001) in seronegative UPIA, directly correlating with GSUS (*R* = 0.36; *p* = 0.02) and PDUS (*R* = 0.55; *p* < 0.001). Synovial CD31^+^ vessels count directly correlated with GSUS (*R* = 0.41; *p* = 0.01) and PDUS (*R* = 0.52; *p* < 0.001). During the follow-up, 6 (14.3%) UPIA reached a definite diagnosis (2 RA, 2 SpA and 2 PsA, respectively). At baseline, UPIA who differentiated had higher GSUS (*p* = 0.01), PDUS scores (*p* = 0.02) and higher histological scores for CD68^+^ (*p* = 0.005 and *p* = 0.04 for lining and sublining respectively), sublining CD3^+^ cells (*p* = 0.002), CD31^+^ vessels count (*p* < 0.001) and higher IL-6 PB levels (*p* = 0.01) than patients who remained as UPIA. MiRNA PCR Array showed that among the 86 tested miRNA species, at baseline, miR-346 and miR-214 were significantly down-regulated (*p* = 0.02 for both) in ST of UPIA who differentiated than in patients who remained as UPIA, inversely correlating with the lining CD68^+^ cells IHC score (*R* = −0.641; *p* = 0.048) and CD31^+^ vessels count (*R* = −0.665; *p* = 0.036) and with higher baseline ST expression of TNF (*p* = 0.014). Finally, logistic regression analysis demonstrated that baseline GSUS and PDUS scores ≥1.5 [OR:22.93 (95%CI:0.98–534.30)] and CD31^+^ vessels count ≥24.3 [OR:23.66 (95%CI:1.50–373.02)] were independent factors associated with the development of definite arthritis.

**Conclusions:** MiRNA signature, histological and US features of ST may help in the identification of seronegative UPIA with high likelihood of clinical differentiation toward definite seronegative arthritis.

## Introduction

Undifferentiated Peripheral Inflammatory Arthritis (UPIA) is a common diagnosis in daily practice at the first clinical evaluation in rheumatological settings. However, the likelihood of developing a well-defined rheumatic disease in UPIA patients as well as the disease progression toward a defined diagnosis, is still matter of debate. Multinational recommendations on the management of UPIA patients have been produced (1), since the future diagnosis and prognosis represent important issues for clinical decision making, including the choice of treatment. Testing for Rheumatoid Factor (RF) and/or anti-citrullinated peptide antibodies (ACPA) should be performed in the evaluation of UPIA patients, as these factors are predictive of Rheumatoid Arthritis (RA) diagnosis and prognosis, although negative tests do not exclude progression to RA (2–4). Ultrasound (US) assessment may be an useful tool in the management of UPIA patients, as the severity of US detected synovitis in UPIA patients was shown to be a predictive factor associated to RA progression (5). However, due to the scarcity of evidences produced so far, its use is not recommended to increase the diagnostic and prognostic power of the clinical examination (6), as well as the use of synovial tissue (ST) biopsy as prognostic tool to foresee the future development of definite diagnosis in UPIA patients (7). In particular, very limited data are available on the diagnostic and prognostic value of synovial tissue biopsy in UPIA patients and regardless to ACPA and RF serological status, synovial CD68 positivity can differentiate among RA, Spondyloarthritis (SpA) and other diagnoses (8).

MicroRNA (miRNA) are small, noncoding, single-stranded RNAs with crucial role in post-transcriptional gene regulation (9), binding to the 3′-untraslated region (3′-UTR) of target messenger RNA (mRNA) and inducing gene silencing by either promoting mRNA degradation or transcript destabilization, resulting in the suppression of target protein (9). Multiple miRNAs have been demonstrated to act as critical epigenetic components in the activation of the immune system in inflammatory joints disease (9–11). To date, limited knowledge is available about miRNAs expression in UPIA patients, with initial data available only about the preclinical phase of seropositive individuals (12, 13).

Based on that, the aims of the study were (i) to define the histological features of ST in terms of CD68^+^, CD21^+^, CD3^+^, CD20^+^ cells, and CD31^+^ vessels of IgA/IgM-RF and ACPA negative, UPIA patients at first clinical examination; (ii) to dissect the possible relation between histological parameters of ST with Gray scale (GS) and Power Doppler (PDUS) scores; (iii) to evaluate possible differences in the epigenetic (miRNA) signature of seronegative UPIA patients based on the differentiation during the follow-up and (iv) to test the prognostic power at baseline, of histological, serological and US parameters of ST inflammation in foreseeing the future achievement of a definite arthritis diagnosis in seronegative UPIA patients.

## Patients and methods

### Study population

Consecutive patients with at least 1 active knee joint with IgA/IgM-RF and ACPA negative UPIA, naive to any Disease Modifying Anti-Rheumatic Drugs (DMARDs), were enrolled in the study at the Division of Rheumatology–Fondazione Policlinico Universitario A. Gemelli, Catholic University of the Sacred Heart. At baseline, demographical, clinical and inflammatory parameters were collected for each patient. Each UPIA patient was tested for IgA and IgM-RF (Orgentec Diagnostika, Bouty, UK) and ACPA (Menarini Diagnostics, Italy) using commercial Enzyme-Linked Immunosorbent Assay (ELISA) and ChemiLuminescence Immunoassay (CLIA) to confirm the inclusion criteria fulfillment. At baseline, each UPIA patient underwent genital and throat swabs to exclude Reactive Arthritis (14), and X-Ray of hands and feet to exclude marginal erosions. Each UPIA patient was then treated with Nonsteroidal Anti-Inflammatory Drugs (NSAIDs) and chloroquine 250 mg/daily and followed every 3 months for 1 year to assess the possible differentiation into known defined arthritis (15–17). In particular, during the follow-up seronegative UPIA patients were re-classified as Rheumatoid Arthritis (RA) if >10 joints (at least 1 small joint) were involved and abnormal C-Reactive Protein (CRP) or abnormal Erythrocyte Sedimentation Rate (ESR) were present (15); as having Psoriatic Arthritis (PsA) if at least 3 points were fulfilled among the following: evidence of psoriasis, presence of psoriatic nail dystrophy, dactylitis, or radiographic evidence of juxta-articular new bone formation on plain radiographs of the hand or foot (16); or as having Spondyloarthritis (SpA) if sacroiliitis was detected on imaging plus one or more SpA features were fulfilled (inflammatory back pain, arthritis, enthesitis, uveitis, dactylitis, psoriasis, inflammatory bowel disease, good response to NSAIDs, family history of SpA, Human Leukocyte Antigen (HLA)-B27 presence or elevated CRP) (17). The study protocol was approved by the local Ethical Committee (Fondazione Policlinico Universitario A. Gemelli, Rome, Italy) and all subjects provided signed informed consent.

### US assessment

At baseline, all UPIA patients underwent US assessment following the same protocol (18). Briefly, each UPIA patient underwent US evaluation using GS and PDUS technique of the following joint sites bilaterally: transverse and longitudinal scanning of dorsal and volar view of the II-III metacarpophalangeal and proximal interphalangeal joints and longitudinal and transverse scanning of the dorsal aspect of the wrist (radiocarpal-intercarpal), bilateral knees and II-V metatarsophalangeal joints. US assessment was performed by one rheumatologist (LP) experienced in US, who was unaware of the clinical and laboratory findings, using a commercially available real-time scanner (MyLabTwice, Esaote) equipped with a multi-frequency linear probe, working at 10–14 MHz. Intra-reader reliability was 0.78. Semi-quantitative scoring methods, which consists of a 0–3 scale, were used to grade the presence of any synovial hypertrophy (SH) on GSUS and its activity (PDUS) for each abovementioned joint, as previously published (18). Briefly, SH was graded on the basis of grayscale images using a semiquantitative scoring method where 0 = no SH, 1 = minimal SH, 2 = moderate SH, and 3 = severe SH, whereas PDUS was recorded using a semiquantitative score where 0 = no PD signal, 1 = minimal PD signal, 2 = moderate PD signal, and 3 = severe PD signal. Two overall SH and PD scores were calculated as the sum of scores obtained from each joint for SH and PD respectively (18).

### CD68, CD3, CD21, CD20, and CD31 synovial tissue expression

At study entry, each UPIA patient underwent ultrasound guided synovial tissue biopsy of the knee. Each tissue was tested through immunohistochemistry for the presence of CD68, CD21, CD3, CD20, and CD31 staining following the already published protocol (19). Slides were examined by two independent evaluators using a light microscope (Leica DM 2000) and all tissues were evaluated using a numerical score based on the number of CD68^+^, CD21^+^, CD3^+^, CD20^+^ cells in the lining and sublining areas of the section (three different fields in each section), with a score of 0 indicating no positive cells; 1 indicating <10% positive cells; 2 indicating 10–50% positive cells; and 3 indicating >50% positive cells. CD31^+^ vessels count was done as mean of the values from three different fields in each section (19). The inter-rater agreement coefficient was assessed for each single IHC marker (Supplementary Table 1).

### MiRNA PCR array on synovial tissue

Total RNA was isolated from synovial tissue of 10 UPIA patients (6 patients who reached a definite diagnosis and 4 patients who remained as UPIA during the follow-up as previously described) using the miRneasy kit (Qiagen). The miScript Reverse Transcription kit (Qiagen) was used for cDNA preparation and the miScript PreAMP PCR kit (Qiagen) was used for cDNA pre-amplification following the manufacturer instructions. miRNA profiling was performed using the Human miScript miRNA PCR array (96 well format from Qiagen-MIHS-111ZA). A set of controls were included on each plate which enabled data analysis using ΔΔCt method of relative quantification, assessment of reverse transcription performance, and assessment of PCR performance. The miScript miRNA PCR array enables SYBR Green-based real-time PCR analysis using Biorad iQ5 real-time PCR system as follows: 95°C for 15 min; 40 cycles of 94°C for 15 s; 55°C for 30 s; and 70°C for 30 s. The relative expression was calculated using the ΔΔCt method (relative gene expression = 2(ΔCt^test^ − ΔCt^control^)] and is presented in fold increase relative to control. The Web-based miScript miRNA PCR array data analysis tool was used to analyze the real-time PCR data (Qiagen).

### TNF expression in whole synovial tissue of UPIA patients by RT-PCR

An iScriptTM cDNA Synthesis Kit (BioRad Laboratories, Hercules, CA) was used for cDNA preparation (10 total UPIA patients: 6 patients who differentiated toward definite seronegative arthritis + 4 patients who remained as UPIA during the follow-up). A FastStart Universal Probe Master (04913949001) (Roche Diagnostics, Germany) was used for RT-PCR using the following primers: human TNF (141083) and Glyceraldehyde 3-phosphate dehydrogenase (GAPDH, 101128) both from Roche Diagnostics, Germany. For the semi-quantitative determination of the expression of human TNF and control GAPDH in whole synovial tissue lysates, ΔCt values were generated after subtraction from the gene of interest (TNF) Ct value of control (GAPDH).

### IL-6, VEGF-A, and VEGF-D ELISA measurement

Each enrolled UPIA patient was tested for Interleukin-6 (IL-6), Vascular Endothelial Growth Factor-A (VEGF-A) and VEGF-D peripheral blood (PB) and synovial fluid (SF) (if available) levels using commercial ELISA kits (all by R&D Systems, United Kingdom). The sensitivity of the test was 0.70 pg/ml for IL-6, 9.0 pg/ml for VEGF-A, and 11.4 pg/ml for VEGF-D respectively.

### Statistical analysis

Statistical analysis was performed using SPSS version 21.0 (SPSS. Chicago. IL-USA) and Prism Software (Graph-Pad, San Diego, CA). Categorical and quantitative variables were described as frequencies, percentage, and mean ± standard deviation (SD). Data on demographic and clinical features were compared between patients by the non-parametric Mann-Whitney *U*-test or χ^2^ test, as appropriate. Spearman's rank correlation test was used for correlation in all analyses. For the miRNA expression profile, data analysis was performed using the supplied software (http://www.qiagen.com/it/shop/genes-and-pathways/data-analysis-center-overview-page/), based on Student's *t*-test of the replicate 2^∧^(−ΔCt) values for each miRNA in the tested group and in the control group. *P* < 0.05 were considered statistically significant. Fold-change values >1 imply an upregulation while fold-change <1 imply down-regulation. using miScript miRNA PCR array, a *t*-test was used to identify significant differences in miRNA expression profiles between UPIA patients who differentiated and those who remained as UPIA during the follow-up. miRNA species with a *p* < 0.05 was considered to be significant. We performed a logistic regression model to determine the influence of the dependent variable “differentiation to defined clinical arthritis” by the independent variables that reached the value of *p* < 0.25 at the univariate analysis. The values are expressed as Odds Ratio (OR) and 95% Confidential Interval (95% CIs), respectively. The Hosmer-Lemeshow test was used to assess the fitting of the model. A *p* < 0.05 was considered statistically significant.

## Results

### Demographical and clinical characteristics of enrolled UPIA patients

Fourty-two UPIA patients were enrolled in the study. Demographical and clinical characteristics of the enrolled UPIA cohort are summarized in Table 1. All UPIA patients were negative for IgA-RF, IgM-RF and ACPA with a mono-oligoarticular pattern of joint involvement. Moreover, none of the patients had evidence of Reactive Arthritis. Among the whole UPIA cohort, 6 (14.3%) patients reached a definite diagnosis during the follow-up (within 6.5 ± 5.4 months) with no differences among the three diagnosis (6.5 ± 7.7 months for RA, 6.5 ± 7.8 months for SpA, and 6.5 ± 4.9 months for PsA respectively). In particular, 2(33.3%) UPIA patients were defined as having RA because of the development of >10 joints involvement and both abnormal CRP and ESR (13), 2(33.3%) as having PsA (due to the subsequent development of psoriasis, psoriatic nail dystrophy and dactylitis) (14) and 2(33.3%) as having SpA (due to the subsequent development of sacroiliitis on imaging) (15), respectively. Moreover, both UPIA patients who differentiated into RA were confirmed as IgA/IgM-RF and ACPA negative at the time of clinical differentiation without developing bone erosions at X-Ray evaluation. UPIA patients reaching a definite diagnosis during the follow-up did not differ from UPIA patients who remained as UPIA afterwards in terms of demographic and clinical [Erythrocyte Sedimentation Rate (ESR), C-Reactive Protein (CRP) and symptoms duration respectively] parameters as well as baseline pharmacological treatment (NSAIDs and corticosteroids usage; Table 1). UPIA patients who differentiated into definite arthritis, had significantly higher baseline IL-6 PB levels (9.27 ± 2.36 pg/ml) compared to patients who remained as UPIA (3.49 ± 4.90 pg/ml; *p* = 0.01), whereas comparable PB VEGF-A and VEGF-D levels were found comparing the two subgroups (Table 1). At baseline, there was direct correlation between IL-6 and CRP PB levels in the whole UPIA cohort (*R* = 0.42; *p* = 0.03). Finally, considering UPIA patients whose SF was available at study entry (*n* = 16) no differences in baseline VEGF-A and VEGF-D SF levels were found, stratifying UPIA patients based on the subsequent differentiation (Table 1 and Supplementary Table 2).

**Table 1:** Demographic, clinical, and inflammatory characteristics of enrolled UPIA patients.

	**Whole UPIA Cohort (*N* = 42)**	**Differentiation**		***p***
		**No (*N* = 36)**	**Yes (*N* = 6)**	
Gender, female *n*(%)	28 (66.7)	24 (66.7)	4 (66.7)	1.00
Age, years (mean ± SD)	53.45 ± 13.89	55.17 ± 12.70	43.17 ± 17.46	0.10
Sympthoms duration, months (mean ± SD)	18.69 ± 16.37	16.69 ± 15.67	19.67 ± 21.43	0.56
ESR, mm/1st hour (mean ± SD)	22.07 ± 19.58	21.06 ± 19.19	28.00 ± 22.63	0.46
CRP, mg/L (mean ± SD)	6.39 ± 11.11	5.27 ± 7.96	12.72 ± 22.10	0.59
Swollen Joint count, (mean ± SD)	2.60 ± 1.86	2.53 ± 1.20	3.11 ± 1.93	0.40
AB positivity, *n*(%)	0 (0%)	–	–	–
Genital swab positivity, *n*(%)	0 (0%)	–	–	–
Throat swab positivity, *n*(%)	0 (0%)	–	–	–
NSAIDs usage, *n*(%)	27 (64.8)	21 (58.3)	6 (100.0)	0.06
GC usage, *n*(%)	9 (21.4)	6 (16.7)	3 (50.0)	0.10
Smoking habit, *n*(%)	14 (33.3)	11 (30.5)	3 (50.0)	0.35
Diagnosis after differentiation				
UPIA ⇒ RA	–	–	2 (33.3)	**–**
UPIA ⇒ PsA	–	–	2 (33.3)	**–**
UPIA ⇒ SpA	–	–	2 (33.3)	**–**
IL-6 (PB), pg/ml (mean ± SD)	4.34 ± 5.04	3.49 ± 4.90	9.27 ± 2.36	**0.01**
IL-6 (SF), pg/ml (mean ± SD)	343.57 ± 167.16	320.67 ± 191.67	389.36 ± 105.81	0.98
VEGF-A (PB), pg/ml (mean ± SD)	42.21 ± 45.91	40.91 ± 45.77	48.49 ± 50.43	0.91
VEGF-D (PB), pg/ml (mean ± SD)	1016.91 ± 502.47	1001.11 ± 454.69	1095.87 ± 747.81	0.88
VEGF-A (SF)^*^, pg/ml (mean ± SD)	1388.68 ± 895.80	1236.92 ± 829.67	1694.21 ± 1130.89	0.38
VEGF-D (SF)^*^, pg/ml (mean ± SD)	338.62 ± 200.40	306.01 ± 186.50	403.82 ± 253.22	0.55

UPIA, Undifferentiated Peripheral Inflammatory Arthritis; ESR, Erytrocyte Sedimentation Rate; CRP, C-Reactive Protein; NSAIDs, Non Steroideal Anti-Inflammatory Drugs; GC, corticosteroids; BMI, Body Mass Index; RA, Rheumatoid Arthritis; PsA, Psoriatic Arthritis; SpA, Spondyloarthritis; IL, interleukin; VEGF, Vascular Endothelial Growth Factor; PB, Peripheral Blood; SF, Synovial Fluid; SD, Standard Deviation;

* *SF was available from 16 UPIA patients (5 UPIA patients who differentiated and 11 UPIA patients who remained as UPIA during the follow-up); bold, p < 0.05 comparing UPIA patients who reached a defined clinical diagnosis during the follow-up (differentiation) vs. patients who remained as UPIA after 1 year follow-up (no differentiation)*.

### Baseline histological features of synovial tissue of UPIA patients are related to the achievement of a defined diagnosis afterwards

**Figure 1:**
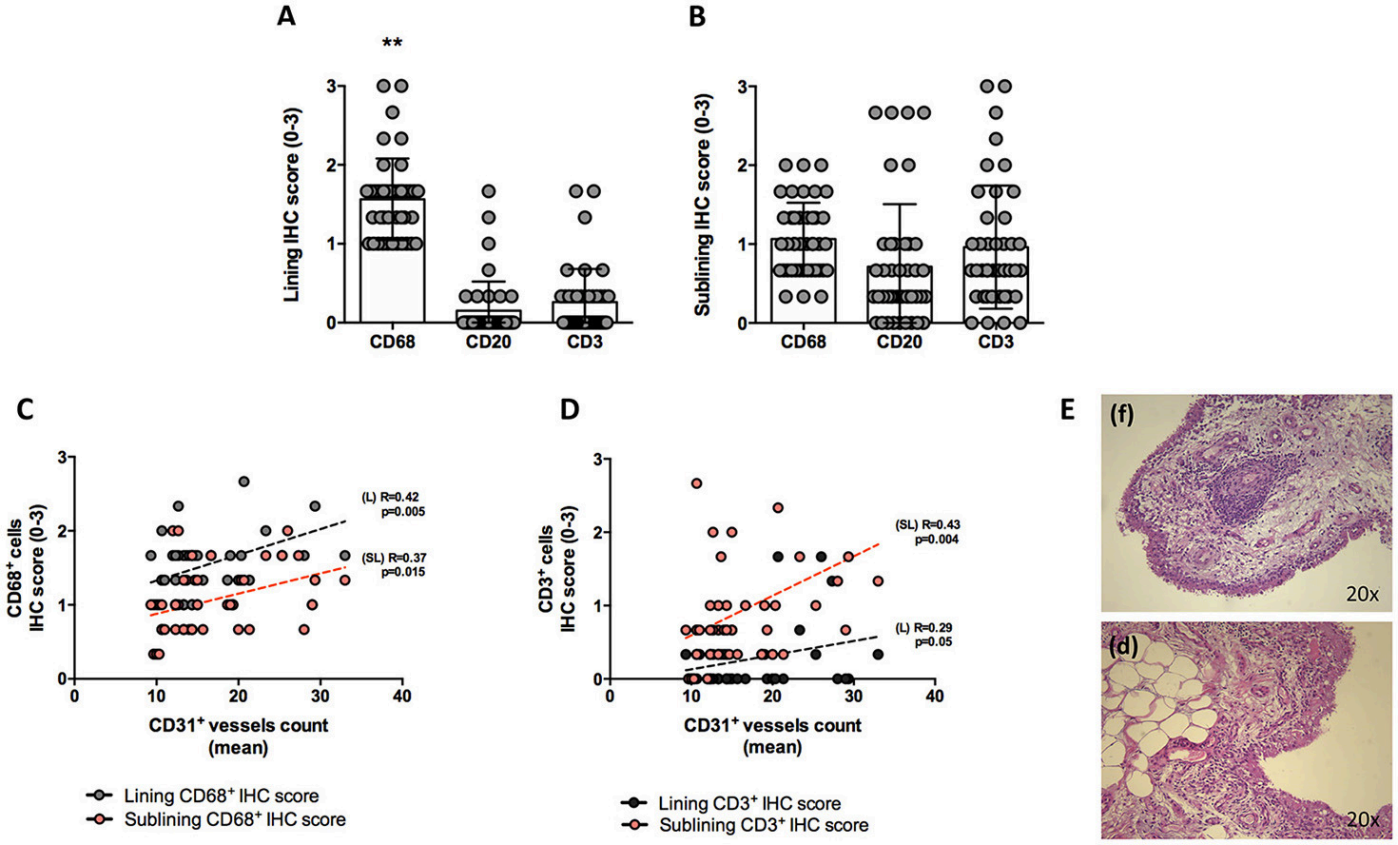
**(A–E)** Synovial tissue immunohistochemical scores for CD68^+^, CD21^+^, CD20^+^, CD3^+^ cells of enrolled seronegative UPIA patients and synovitis pathotype distribution. **(A,B)** Baseline lining and sublining IHC scores for CD68^+^, CD20^+^ and CD3^+^ cells of synovial tissue of UPIA patients cohort (**p < 0.001 ANOVA Test); Mann-Whitney test for all comparisons: lining CD68^+^ vs lining CD20^+^ cells (1.57 ± 0.52 vs. 0.12 ± 0.40; *p* < 0.001) and lining CD68^+^ cells vs lining CD3^+^ cells (1.57 ± 0.52 vs. 0.25 ± 0.42; *p* < 0.001); CD20^+^ cells vs lining CD3^+^ cells (0.12 ± 0.40 vs. 0.25 ± 0.42; *p* = 0.13) (Data on graphs represent mean ± SD); **(C)** Correlation between lining and sublining ST IHC scores of CD68^+^ cells and CD31^+^ vessels count in the whole cohort of seronegative UPIA patients; **(D)** Correlation between lining and sublining ST IHC scores of CD3^+^ cells and CD31^+^ vessels count in the whole cohort of seronegative UPIA patients; **(E)** Example photos of H&E staining of ST of UPIA patients with follicular **(f)** or diffuse **(d)** synovitis pattern (magnification 20x). UPIA, Undifferentiated Peripheral Inflammatory Arthritis; CD, Cluster designation; IHC, Immunohistochemistry; ST, Synovial Tissue; L, Lining; SL, Sublining; H&E, Haematoxylin and Eosin; SD, Standard Deviation.

**Figure 2:**
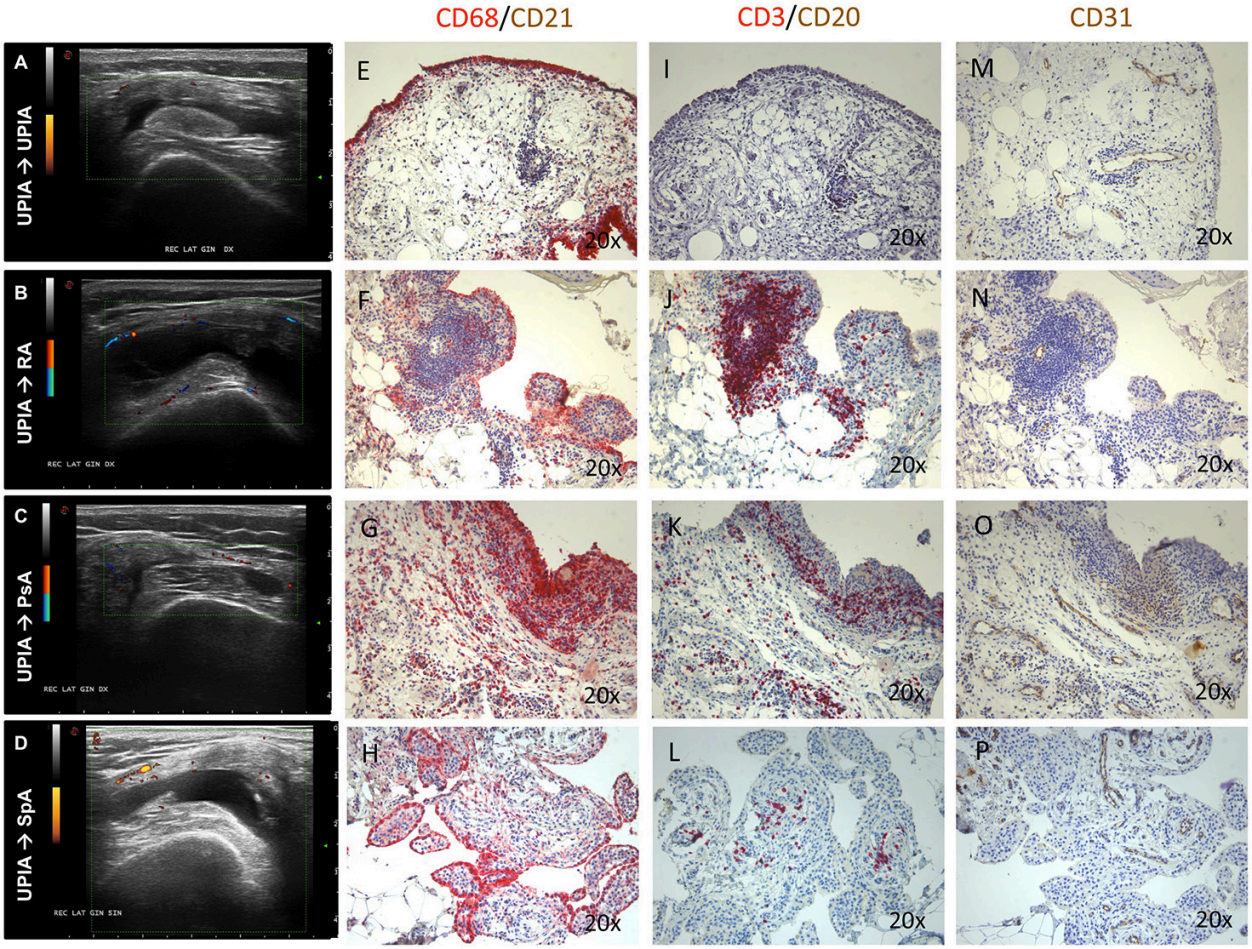
**(A–P)** Ultrasound assessment and immunohistochemistry for CD68^+^, CD21^+^, CD20^+^, CD3^+^ cells and CD31^+^ vessels of synovial tissue of enrolled UPIA patients based on the clinical differentiation. **(A)** Example photo of the ultrasound assessment of knee joint of UPIA patient who remained as UPIA afterwards; **(B)** Example photo of the ultrasound assessment of knee joint of UPIA patient who differentiated into RA afterwards; **(C)** Example photo of the ultrasound assessment of knee joint of UPIA patient who differentiated into PsA afterwards; **(D)** Example photo of the ultrasound assessment of knee joint of UPIA patient who differentiated into SpA afterwards; Example photos of CD68 (Red)/CD21 (Brown) staining of ST biopsies from UPIA patients who **(E)** remained as UPIA, **(F)** differentiated into RA, **(G)** differentiated into PsA or **(H)** differentiated into SpA afterwards (Magnification 20X); Example photos of CD3 (Red)/CD20 (Brown) staining of ST biopsies from UPIA patient who **(I)** remained as UPIA, **(J)** differentiated into RA, **(K)** differentiated into PsA or **(L)** differentiated into SpA afterwards (Magnification 20X); Example photos of CD31 (Brown) staining of ST biopsies from UPIA patient who **(M)** remained as UPIA, **(N)** differentiated into RA, **(O)** differentiated into PsA or **(P)** differentiated into SpA afterwards (Magnification 20X); US picture with PD scale of the knee used for ST biopsy is shown next to the corresponding patient; ST, synovial tissue; UPIA, Undifferentiated Peripheral Inflammatory Arthritis; CD, Cluster designation; RA, Rheumatoid Arthritis; SpA, Spondyloarthritis; PsA, Psoriatic Arthritis.

**Figure 3:**
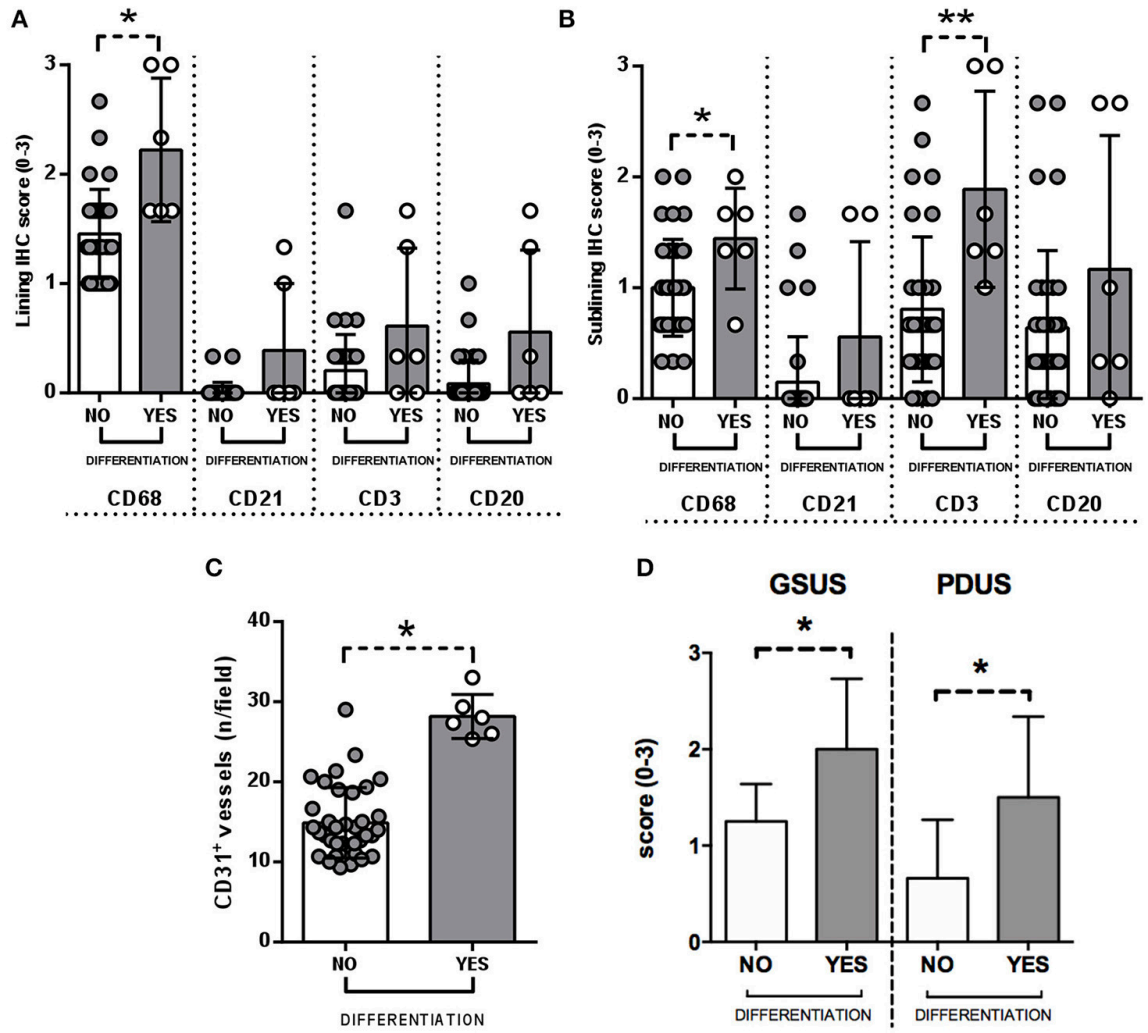
**(A–D)** Immunohistochemistry scores of synovial tissue of UPIA patients based on the differentiation during the follow-up. **(A)** Lining IHC scores for CD68^+^, CD21^+^, CD3^+^, and CD20^+^ cells in ST of UPIA patients based on the differentiation during the follow-up; *p = 0.005 lining CD68^+^ cells IHC score comparing UPIA patients who differentiated compared to patients who remained as UPIA during the follow-up; **(B)** Sublining IHC scores for CD68^+^, CD21^+^, CD3^+^, and CD20^+^ cells in ST of UPIA patients based on the differentiation during the follow-up; *p = 0.04 sublining CD68^+^ cells IHC score comparing UPIA patients who differentiated vs. patients who remained as UPIA during the follow-up; **p = 0.002 sublining CD3^+^ cells IHC score comparing UPIA patients who differentiated vs. patients who remained as UPIA during the follow-up; **(C)** IHC scores for CD31^+^ vessels in ST of UPIA patients based on the differentiation during the follow-up; *p < 0.001 CD31^+^ vessels count comparing UPIA patients who differentiated vs patients who remained as UPIA during the follow-up; **(D)** Baseline GSUS and PDUS scores in seronegative UPIA patients who reached a definite diagnosis during the follow-up compared to UPIA patients who remained as UPIA (*p < 0.001 for GSUS and *p = 0.01 for PDUS respectively); (All data on graphs represent mean ± SD); IHC, Immunohistochemistry; CD, Cluster Designation; UPIA, Undifferentiated Peripheral Inflammatory Arthritis; SD, Standard Deviation.

At baseline, each enrolled UPIA patient underwent ST biopsy of the knee which was tested through immunohistochemistry for the presence of CD68^+^, CD21^+^, CD3^+^, CD20^+^ cells, and CD31^+^ vessels count showing that CD68^+^ cells represent the most common inflammatory cells (*p* < 0.001) in the lining of the synovial tissue of seronegative UPIA patients (Figure 1A) whereas a comparable distribution was found in the sublining area concerning the analyzed inflammatory cells (*p* = 0.06; Figure 1B). Among the assessed inflammatory cells, the IHC scores for CD68^+^ and CD3^+^ cells, in ST of seronegative UPIA patients, directly correlates with the entity of tissue vasculature, expressed by the mean number of CD31^+^ vessels (*R* = 0.42; *p* = 0.005 and *R* = 0.37; *p* = 0.015 for lining and sublining IHC scores for CD68^+^ cells; *R* = 0.29 *p* = 0.05 and *R* = 0.43; *p* = 0.004 for lining and sublining IHC scores for CD3^+^ cells; Figures 1C,D) whereas no significant correlation was found between lining and sublining IHC scores for CD20^+^ cells and the mean number of CD31^+^ vessels (*R* = 0.25, *p* = 0.12, and *R* = 0.21, *p* = 0.18 for lining and sublining CD20^+^ cells respectively). Considering the synovial tissue pattern in the whole cohort, a follicular synovitis was found in 6(14.3%) seronegative UPIA patients without significant difference stratifying UPIA patients according to the subsequent differentiation [2(33.3%) of UPIA patients who differentiated into definite arthritis had baseline follicular synovitis vs 4(11.1%) UPIA patients who remained as UPIA had baseline follicular synovitis (*p* = 0.14)] (Figure 1E). However, analyzing the distribution of synovial tissue resident inflammatory cells at baseline (Figures 2A–P), significantly higher histological scores for lining CD68^+^ cells (2.22 ± 0.65 vs. 1.45 ± 0.41, *p* = 0.005; Figure 3A), sublining CD68^+^ cells (1.44 ± 0.45 vs. 1.00 ± 0.44, *p* = 0.04), sublining CD3^+^ cells (1.88 ± 0.89 vs. 0.81 ± 0.65, *p* = 0.002; Figure 3B) and higher CD31^+^ vessels count (28.17 ± 2.76 vs. 14.88 ± 7.39, *p* < 0.001; Figure 3C) were found in UPIA patients who differentiated into defined seronegative arthritis than patients who remained as UPIA during the follow-up. Interestingly, both UPIA patients who differentiated into RA had follicular synovitis at baseline compared to none of UPIA patients who differentiated into PsA or SpA.

### Baseline GSUS and PDUS scores are associated with clinical differentiation and directly correlate with histological scores of synovial resident inflammatory cells in UPIA patients

The US assessment showed that enrolled seronegative UPIA patients showed GSUS of and PDUS scores of 1.36 ± 0.77 and 1.45 ± 0.88 respectively. Moreover, at baseline, UPIA patients who reached a definite diagnosis during the follow-up, had significantly higher GSUS (2.00 ± 0.63) and PDUS scores (1.50 ± 0.84) compared to patients who remained as UPIA afterwards (1.25 ± 0.44 for GSUS score, *p* = 0.01; and 0.58 ± 0.60 for PDUS score respectively, *p* = 0.02; Figure 3D). Moreover, at baseline, GSUS and PDUS scores directly correlated with ST histological features in terms of synovial resident inflammatory cells. In particular, baseline GSUS score directly correlated with CD68^+^ lining (*R* = 0.55; *p* < 0.001) and sublining (*R* = 0.37; *p* = 0.02) scores, CD3^+^ sublining score (*R* = 0.47; *p* = 0.002) and CD31^+^ vessels count (*R* = 0.41; *p* = 0.01). Similarly, baseline PDUS score directly correlated with CD68^+^ lining (*R* = 0.36; *p* = 0.02) and sublining (*R* = 0.33; *p* = 0.03) scores, CD3^+^ sublining score (*R* = 0.47; *p* = 0.002) and CD31^+^ vessels count (*R* = 0.52; *p* < 0.001; Supplementary Figure 1).

### Whole synovial tissue miRNA expression profile is altered in seronegative UPIA patients based on the clinical differentiation and is related to TNF expression and histological features of synovial tissue

**Figure 4:**
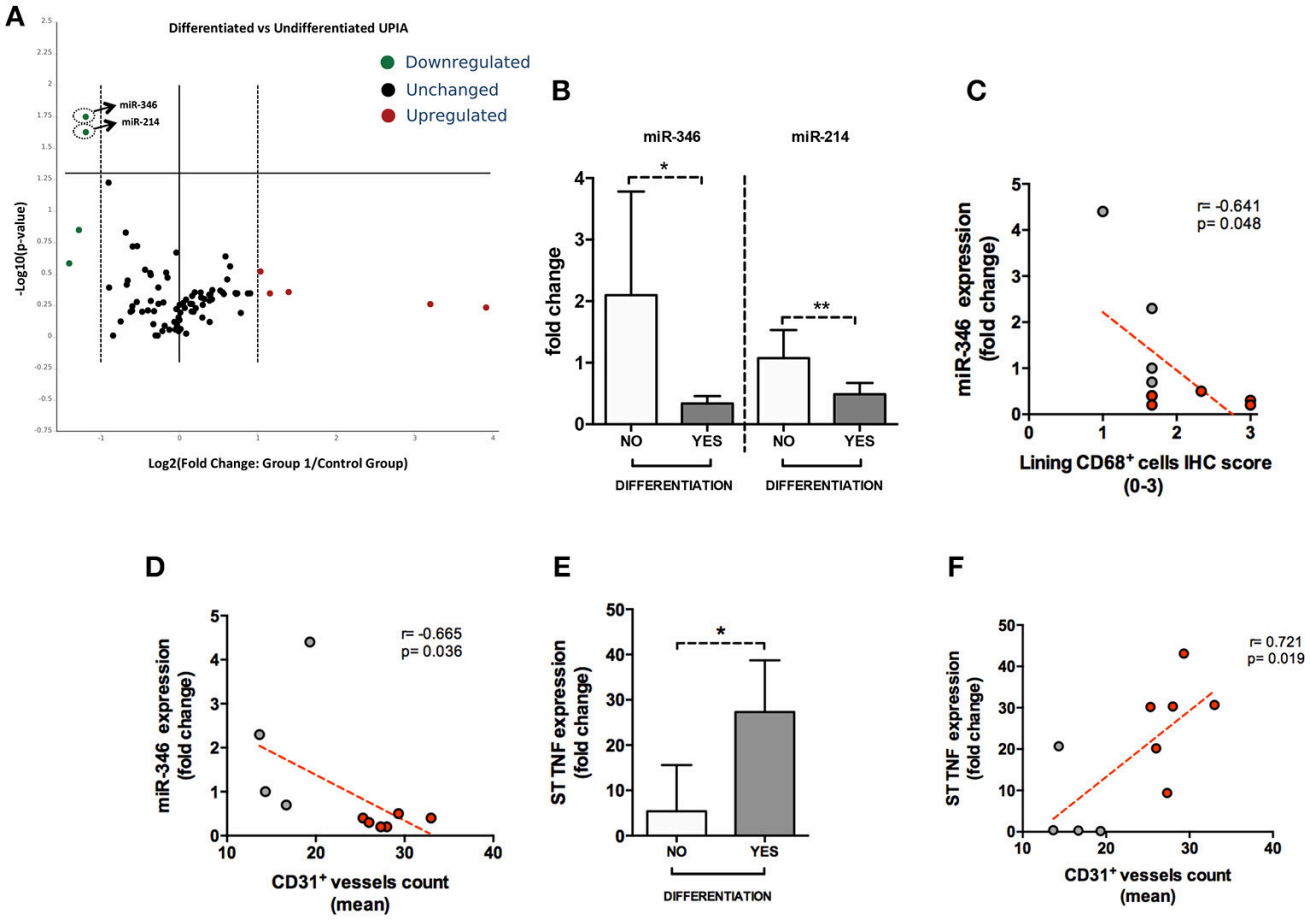
**(A–F)** microRNA panel of the whole synovial tissue of UPIA patients based on the clinical differentiation during the follow-up and its correlations with TNF tissue expression and histological features. **(A)** Volcano plot showing expression of miRNAs in the whole synovial tissue of UPIA patients; Color scheme: green decreased expression; Red, Increased expression; Black, Unchanged. The miRNAs that are significantly altered between the two groups are located above the horizontal black line corresponding to *p* < 0.05; **(B)** miR-346 and miR-214 expression in whole synovial tissue of seronegative UPIA stratified based on the clinical differentiation during the follow-up (*p = 0.02; **p = 0.02) (Data on graphs represent mean ± SD); **(C)** Correlation between miR-346 expression in the whole synovial tissue of UPIA patients and lining CD68^+^ cells IHC semi-quantitative score; Red, UPIA patients who differentiated during the follow-up; Gray, UPIA patients who remained as UPIA during the follow-up; **(D)** Correlation between miR-346 expression in the whole synovial tissue of UPIA patients and CD31^+^ vessels count (mean); Red, UPIA patients who differentiated during the follow-up; Gray, UPIA patients who remained as UPIA during the follow-up; **(E)** TNF expression in whole synovial tissue of seronegative UPIA stratified based on the clinical differentiation during the follow-up (*p = 0.01) (Data on graphs represent mean ± SD); **(F)** Correlation between TNF expression in the whole synovial tissue of UPIA patients and CD31^+^ vessels count (mean); Red, UPIA patients who differentiated during the follow-up; Gray, UPIA patients who remained as UPIA during the follow-up; SD, Standard Deviation; CD, Cluster Designation; UPIA, Undifferentiated Peripheral Inflammatory Arthritis.

To assess if seronegative UPIA patients differ in terms of the whole synovial tissue epigenetic signature, based on the clinical differentiation likelihood, we analyzed, in the synovial tissue, the expression of a panel of miRNAs selected on the basis of their relevance in the regulation of inflammatory cells activation (Supplementary Figure 2). Overall, our analysis showed that the expression of miR-346 and miR-214 was significantly decreased in UPIA patients who reached a definite diagnosis during the follow-up compared to UPIA patients who remained as UPIA (0.44 fold change; *p* = 0.017 and 0.44 fold change; *p* = 0.023 for miR-346 and miR-214 respectively; Table 2). As shown in Figure 4A, Volcano scatter plot showed that miR-346 and miR-214 were the most down-regulated miRNAs (upper left corner of the plot) in UPIA patients who differentiated during the follow-up than UPIA patients who remained as UPIA. In particular, as shown in Figure 4B, UPIA patients who differentiated during the follow-up showed, at baseline, significantly lower expression of miR-346 and miR-214 at synovial tissue level compared to UPIA patients who remained as UPIA (*p* = 0.017 and *p* = 0.023 for miR-346 and miR-214 respectively). Moreover, at baseline miR-346 expression in the whole synovial tissue inversely correlated with the lining CD68^+^ cells IHC score (*R* = −0.64; *p* = 0.04; Figure 4C) and with the synovial tissue CD31^+^ vessels count (*R* = −0.66; *p* = 0.03) in seronegative UPIA patients (Figure 4D). Finally, as shown in Figure 4E, UPIA patients who differentiated during the follow-up showed higher baseline expression of TNF in the synovial tissue than patients who remained as UPIA (*p* = 0.01), directly correlating with the baseline synovial tissue CD31^+^ vessels count (*R* = 0.72; *p* = 0.02; Figure 4F).

**Table 2:** Baseline fold change expression of microRNAs in whole synovial tissue comparing seronegative UPIA patients who achieved diagnosis of definite seronegative arthritis vs. UPIA patients who remained as UPIA during the follow-up.

	**Diff UPIA vs. no-Diff UPIA**	
**miRNA**	**Fold change**	***P*-Value**
hsa-let-7a-5p	1.72	0.64274
hsa-let-7b-5p	15.06	0.58211
hsa-let-7c-5p	1.06	0.938401
hsa-let-7d-5p	1.16	0.589124
hsa-let-7e-5p	0.83	0.404177
hsa-let-7f-5p	1.31	0.75907
hsa-let-7g-5p	1.53	0.348485
hsa-let-7i-5p	1.01	0.637933
hsa-miR-100-5p	0.92	0.873711
hsa-miR-101-3p	2.23	0.449745
hsa-miR-106b-5p	1.31	0.460063
hsa-miR-125b-5p	9.19	0.54778
hsa-miR-126-3p	1.00	0.731593
hsa-miR-128-3p	1.12	0.625592
hsa-miR-130b-3p	0.76	0.61418
hsa-miR-132-3p	0.63	0.383393
hsa-miR-139-5p	0.63	0.355359
hsa-miR-142-3p	2.63	0.438817
hsa-miR-142-5p	1.15	0.441401
hsa-miR-145-5p	0.80	0.78913
hsa-miR-146a-5p	0.99	0.699398
hsa-miR-146b-5p	1.57	0.274573
hsa-miR-147a	0.90	0.337146
hsa-miR-148a-3p	0.97	0.213462
hsa-miR-150-5p	1.50	0.228892
hsa-miR-155-5p	0.83	0.544259
hsa-miR-15a-5p	1.06	0.504269
hsa-miR-15a-3p	0.69	0.526566
hsa-miR-15b-5p	2.05	0.301488
hsa-miR-16-5p	1.83	0.450233
hsa-miR-17-5p	1.12	0.471384
hsa-miR-17-3p	1.00	0.554158
hsa-miR-181a-5p	0.66	0.616177
hsa-miR-181b-5p	0.69	0.189542
hsa-miR-181c-5p	0.65	0.629276
hsa-miR-181d-5p	0.54	0.05944
hsa-miR-182-5p	1.47	0.441483
hsa-miR-184	0.41	0.141154
hsa-miR-18a-5p	1.65	0.454234
hsa-miR-191-5p	1.31	0.513057
hsa-miR-195-5p	1.87	0.449673
hsa-miR-199a-5p	0.60	0.751729
hsa-miR-19a-3p	1.64	0.447654
hsa-miR-19b-3p	1.67	0.449978
hsa-miR-204-5p	0.38	0.25995
hsa-miR-20a-5p	1.32	0.457404
hsa-miR-20b-5p	1.48	0.455427
hsa-miR-21-5p	0.66	0.569126
hsa-miR-210-3p	0.81	0.971131
**hsa-miR-214-3p**	**0.44**	**0.023626**
hsa-miR-221-3p	0.88	0.813492
hsa-miR-222-3p	0.74	0.292096
hsa-miR-223-3p	1.33	0.501084
hsa-miR-23a-3p	0.99	0.897427
hsa-miR-23b-3p	0.77	0.310103
hsa-miR-24-3p	1.23	0.699806
hsa-miR-25-3p	0.98	0.80932
hsa-miR-26a-5p	1.25	0.498431
hsa-miR-26b-5p	1.21	0.442715
hsa-miR-27a-3p	1.13	0.628788
hsa-miR-27b-3p	0.97	0.871495
hsa-miR-28-5p	0.78	0.516299
hsa-miR-29a-3p	1.01	0.862774
hsa-miR-29b-3p	1.30	0.460232
hsa-miR-29c-3p	1.23	0.55443
hsa-miR-30a-5p	1.05	0.586439
hsa-miR-30b-5p	1.03	0.537262
hsa-miR-30c-5p	1.34	0.423138
hsa-miR-30d-5p	0.97	0.598599
hsa-miR-30e-5p	1.10	0.544092
hsa-miR-31-5p	0.89	0.307602
hsa-miR-326	0.80	0.622988
hsa-miR-331-3p	0.54	0.40421
hsa-miR-335-5p	0.96	0.75784
hsa-miR-342-3p	1.12	0.545177
**hsa-miR-346**	**0.44**	**0.017843**
hsa-miR-34a-5p	0.56	0.973224
hsa-miR-365b-3p	0.87	0.530566
hsa-miR-423-5p	0.72	0.62926
hsa-miR-574-3p	0.86	0.895815
hsa-miR-92a-3p	1.44	0.427582
hsa-miR-93-5p	1.21	0.484929
hsa-miR-98-5p	0.62	0.148112
hsa-miR-99a-5p	0.83	0.971675

*miRNA profiling was analysed in the whole synovial tissue total RNA samples obtained from seronegative UPIA patients z(6 UPIA patients who reached a definite diagnosis of seronegative arthritis during the follow-up and 4 UPIA patients who remained as UPIA); Columns highlighted in bold represent miRNAs that show significant differential expression between the two groups (p < 0.05); Red-highlighted values indicate up-regulated miRNA species whereas green-highlighted values indicate down-regulated miRNA species in ST; MiRNA, microRNA*.

### Baseline GSUS and PDUS scores and CD31^+^ vessels count are independent factors associated with clinical differentiation in UPIA patients

**Figure 5:**
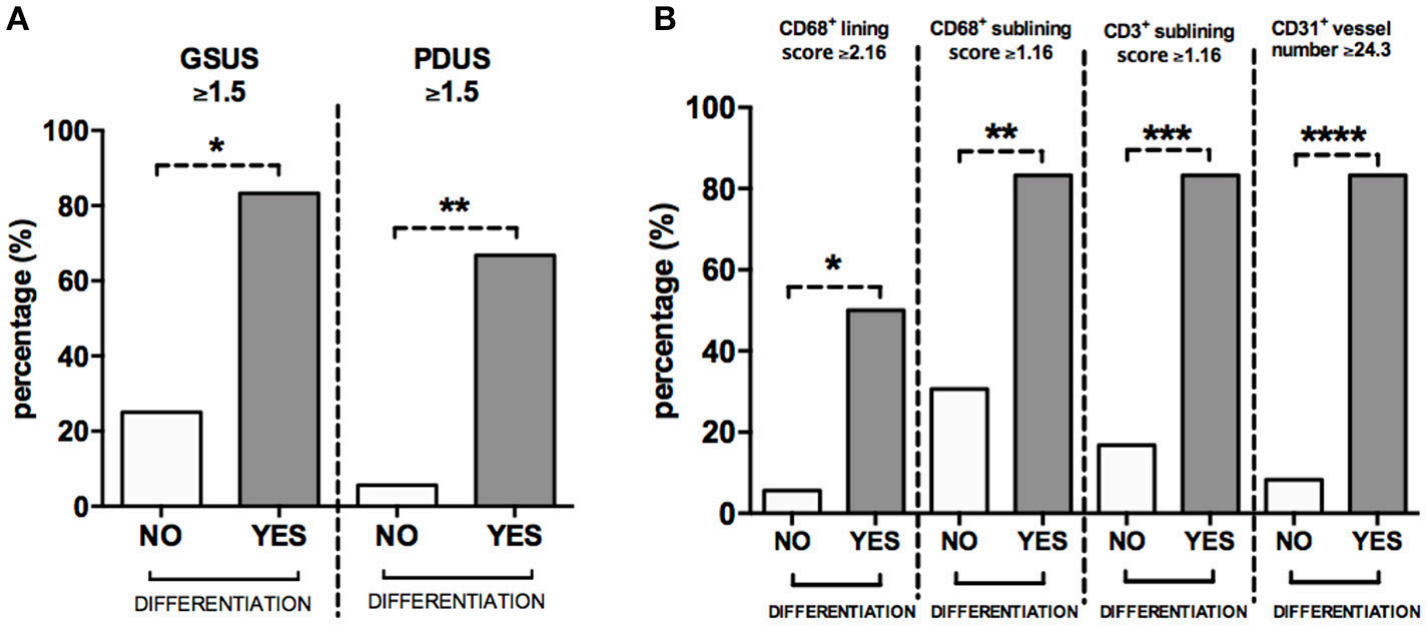
**(A,B)** Baseline GSUS, PDUS and immunohistochemistry cut-off values for differentiation into seronegative definite arthritis in UPIA patients. **(A)** Distribution of GSUS and PDUS cut-off values in UPIA patients based on the differentiation during the follow-up; 5(83.3%) UPIA patients who reached a defined diagnosis during the follow-up had baseline GSUS score ≥1.5 vs. 9(25.0%) patients who remained as UPIA during the follow-up had baseline GSUS score ≥1.5, *p = 0.01; 4(66.7%) UPIA patients who reached a defined diagnosis during the follow-up had baseline PDUS score ≥1.5 vs. 2(5.6%) patients who remained as UPIA during the follow-up had baseline PDUS score ≥1.5, *p = 0.002; **(B)** Distribution of IHC cut-off values in UPIA patients based on the differentiation during the follow-up; 3(50.0%) UPIA patients who reached a defined diagnosis during the follow-up had baseline lining CD68^+^ cells score ≥2.16 vs. 2(5.6%) patients who remained as UPIA during the follow-up had baseline lining CD68^+^ cells score ≥2.16, *p = 0.01; 5(83.3%) UPIA patients who reached a defined diagnosis during the follow-up had baseline sublining CD68^+^ cells score ≥1.16 vs. 11(30.6%) patients who remained as UPIA during the follow-up had baseline lining CD68^+^ cells score ≥1.16, **p = 0.02; 5(83.3%) UPIA patients who reached a defined diagnosis during the follow-up had baseline sublining CD3^+^ cells score ≥1.16 vs. 6(16.7%) patients who remained as UPIA during the follow-up had baseline sublining CD3^+^ cells score ≥1.16, ***p = 0.003; 5(83.3%) UPIA patients who reached a defined diagnosis during the follow-up had baseline CD31^+^ vessels count ≥24.3 vs. 3(8.3%) patients who remained as UPIA during the follow-up had baseline CD31^+^ vessels count ≥24.3, ****p < 0.001. Data are shown as percentage of UPIA patients fulfilling the different cut-off values of US parameters or IHC scores, based on their clinical differentiation during the follow-up (all data on graphs represent mean ± SD). GSUS, Gray Scale Ultrasonography; PDUS, Power Doppler Ultrasonography; IHC, Immunohistochemistry; CD, Cluster Designation; UPIA, Undifferentiated Peripheral Inflammatory Arthritis; SD, Standard Deviation.

To define the best cut-off value for CD68^+^ cells lining and sublining scores, CD3^+^ cells sublining score, CD31^+^ vessels count and IL-6 PB levels, able to identify UPIA patients who reached a defined diagnosis during the follow-up, ROC analysis was performed for each parameter (Supplementary Table 3). UPIA patients who reached a definite diagnosis during the follow-up had more likely baseline GSUS score ≥1.5 (83.3%) and PDUS score ≥1.5 (66.7%) than UPIA patients who remained as UPIA (25.0% patients with GSUS ≥1.5, *p* = 0.01 and 5.6% patients with PDUS ≥1.5, *p* = 0.002; Figure 5A). As shown in Figure 5B, UPIA patients who reached a definite diagnosis during the follow-up had more baseline lining CD68^+^ cells score ≥2.16 (50.0%), sublining CD68^+^ cells score ≥1.16 (83.3%), sublining CD3^+^ cells score ≥1.16 (83.3%), and CD31^+^ vessels count ≥24.3 (83.3%) compared to patients who remained as UPIA (5.6% patients with lining CD68^+^ cells score ≥2.16, *p* = 0.01; 30.6% patients with sublining CD68^+^ cells score ≥1.16, *p* = 0.02; 16.7% patients with sublining CD3^+^ cells score ≥1.16, *p* = 0.003 and 8.3% patients with CD31^+^ vessels score ≥24.3, *p* < 0.001). Moreover, UPIA patients who differentiated within 1 year, had more likely IL-6 PB levels ≥4.75 pg/ml (100.0%) than UPIA patients who remained as UPIA (21.7%; *p* = 0.01). Finally, the logistic regression analysis (data not shown) demonstrated that having baseline GSUS and PDUS scores ≥1.5 at US assessment [OR: 22.93 (95%CI:0.98–534.31)] and CD31^+^ vessels count ≥24.3 [OR: 23.66 (95%CI: 1.50–373.00)] are two independent factors associated with future achievement of defined arthritis in seronegative UPIA patients (Hosmer-Lemeshowtest: *p* = 0.35).

## Discussion

In daily practice, a large amount of patients who present with recent-onset arthritis have UPIA at the first clinical examination, however there is still an unmet medical need in the identification of possible diagnostic and prognostic biomarkers to be applied in such patients. In the current study, we found that ST of seronegative UPIA patients show specific microRNA profiling based on the likelihood chance of clinical future differentiation. Moreover, we demonstrated that the combined analysis of histological and US features of ST, at the first clinical examination, may help in the identification of UPIA patients with higher chance of further differentiation.

As known, ST biopsy is not routinely performed in clinical practice (20), since often, history-taking, physical and radiographic examination, as well as serum and autoimmune markers are sufficient to make a defined diagnosis (1). However, autoantibody negativity may increase the difficulty in reaching a definite classification. Therefore, in our study we included only IgA/IgM-RF and ACPA negative UPIA patients to assess the possible prognostic role of ST biopsy in foreseeing the future clinical development into defined arthritis. Kraan et al. previously demonstrated that immunohistochemical analysis of synovial tissue from patients with early undifferentiated arthritis may help in the differentiation of non-RA from RA patients with increased plasmacells, B cells and macrophages infiltration in the latter group (7). In our study, we found that CD68^+^ cells represent the most abundant inflammatory cells within the ST with a direct correlation with US parameters in seronegative UPIA. Moreover, we found that seronegative UPIA patients who differentiate into further defined arthritis, show higher expression of lining and sublining CD68^+^ cells, sublining CD3^+^ cells, increased number of CD31^+^ vessels and higher IL-6 PB levels than patients who remain as UPIA during the follow-up. Among different immunohistochemical parameters, logistic regression analysis showed that having a CD31^+^ vessels count ≥24.3 is an independent factor associated with further differentiation in seronegative UPIA patients. To avoid therapeutic bias, in the present study we enrolled only UPIA patients taking NSAIDs or corticosteroids compared to the study by Kraan and colleagues (7), in which DMARDs including antimalarials, sulphasalazine and gold were allowed.

The use of US in UPIA patients assessment is not routinely recommended by experts for diagnostic and prognostic purposes (1), despite US may offer advantages, being more sensitive than physical examination and X-rays. To date, few studies revealed that PDUS and GSUS parameters may be considered potential candidates in UPIA patients examination (6). Recently, Horton SC, et al investigated the power of US assessment in UPIA patients at risk of RA development, showing that (9/42) UPIA patients progressed toward definite diagnosis of RA during 1 year follow-up and that GSUS synovitis was a predictive factor of disease progression (5). In our study we assessed, for the first time, the prognostic role of US in combination with ST biopsy in foreseeing the future differentiation into defined arthritis in seronegative UPIA patients showing that, at baseline, PDUS and GSUS scores are directly associated with histological synovial inflammation in terms of CD68^+^, CD3^+^ cells and CD31^+^ vessels. Moreover, logistic regression analysis revealed that in seronegative UPIA patients, having at baseline PDUS and GSUS scores ≥1.5, is an independent factor associated with clinical differentiation afterwards, increasing the evidence supporting the utility of US assessment in the management of seronegative UPIA patients. These results strengthen the link between US detected synovitis and histology supporting its feasible use in the clinical management of UPIA patients.

The analysis of vascular morphology seemed to increase the classification power to distinguish between RA (with straight pattern) from PsA or SpA patients (with tortuous pattern) at arthroscopic evaluation (21). Therefore, since the angiogenic processes play crucial role in the initiation and perpetuation of synovial inflammation, allowing the influx of inflammatory cells in ST (22, 23), VEGF-A and VEGF-D plasma and synovial fluid levels were tested. We found that baseline VEGF-A and VEGF-D plasma and synovial fluid levels are not different in UPIA patients who reach a definite diagnosis compared to patients who remain as UPIA confirming previous data in a smaller study population (24). The number of patients who evolved into RA, PsA or SpA was insufficient to determine possible differences among the three diseases. Yet the mean number of CD31^+^ vessels in ST, along with US parameters appear to be the most efficient way to identify UPIA patients with high likelihood to differentiate into a definite chronic seronegative arthritis.

To date, few data have been produced on the role of miRNA as predictive tools in arthritis development and prognosis, with scarce studies only assessing miRNAs profile in peripheral blood derived cells of seropositive individuals at risk of arthritis development (13) with no data on ST. In our knowledge, this is the first study analyzing the expression of a panel of different miRNA species in the whole synovial tissue of ACPA/IgA-IgM negative UPIA patients stratified based on their clinical differentiation toward definite seronegative arthritis within 1 year follow-up. Among the assessed miRNAs, miR-346, and miR-214 arose to be significantly repressed, at baseline, in ST of seronegative UPIA patients who differentiated compared to patients who remained as UPIA during the follow-up. To date, very limited data were produced on the effect of miR-214 downregulation on the pathogenesis of arthritis. Lai NS et al recently found that TNF-α stimulation is able to significantly reduce miR-214 expression from T cells *in vitro* and that the transfection with miR-214 mimic conversely suppresses TNF-α signal, providing a survival signal for T cells through the ERK/JNK pathway (25–27). MiR-346 has been demonstrated to play a role in the control of the inflammatory response acting as a positive regulator of Tristetraprolin, a RNA binding protein that destabilizes TNF mRNA, acting as a negative regulator of TNF synthesis (28). Moreover, miR-346 was found to be involved in the regulation of Bruton Tyrosine Kinase involved in the TNF release in RA (29, 30). In our cohort of seronegative UPIA patients, the expression of miR-346 in ST inversely correlates with synovial IHC scores of CD68^+^ cells and CD31^+^ vessels count, suggesting a possible link between miR-346 downregulation and ST inflammation in terms of immune cells infiltrate and vascularity. Moreover, we found that seronegative UPIA patients who achieved a definite diagnosis during the follow-up showed, at baseline, higher expression of TNF at ST level, supporting the notion that seronegative UPIA patients with higher risk of clinical differentiation may have more stable TNF mRNA due to miR-346 downregulation (28). This hypothesis is supported by the finding that the TNF expression in the ST directly correlates, at baseline, with the entity of the tissue vasculature (expressed by the CD31^+^ vessels count) which enables inflammatory cells ST influx.

In conclusion, we believe that this study results may have an impact for the daily management of seronegative UPIA patients as supported by the direct association between synovial membrane US features and histologically proven tissue inflammation associated with their chance of future differentiation. Moreover, despite mono or oligoarticular joint involvement, seronegative UPIA patients with high likelihood of clinical differentiation are characterized by aberrant microRNA signature at ST level with a direct correlation with histological and US features of ST supporting the need for a more aggressive treatment.

## Author contributions

SA, GF, and EG: conceived the study; SA, LP, MG, AF, EG, and FF: collected clinical data; LP: performed US assessment; SA, BT, LB, CD, GD, and RB: performed experiments; SA, LP, and BT: performed statistical analysis; SA, BT, LP, LB, CD, MG, GD, RB, AF, FF, EG, and GF: drafted and revised the manuscript. All authors read and approved the final manuscript.

### Conflict of interest statement

The authors declare that the research was conducted in the absence of any commercial or financial relationships that could be construed as a potential conflict of interest.
