# Supplementary material for: Synovial Predictors of Differentiation to Definite Arthritis in Patients With Seronegative Undifferentiated Peripheral Inflammatory Arthritis: microRNA Signature, Histological, and Ultrasound Features
Source: Front Med (Lausanne). 2018 Jul 3;5:186. doi: 10.3389/fmed.2018.00186 (PMC6037719; doi:10.3389/fmed.2018.00186)
Supplement: Supplementary file 1 [file Table_1.doc]

Supplementary Material

**Synovial predictors of differentiation to definite arthritis in patients with seronegative undifferentiated peripheral inflammatory arthritis: microRNA signature, histological and ultrasound features.**

Stefano Alivernini^1^, Barbara Tolusso^1^, Luca Petricca^1^, Laura Bui^2^, Clara Di Mario^1^, Maria Rita Gigante^1^, Gabriele Di Sante^1^, Roberta Benvenuto^2^, Anna Laura Fedele^1^, Francesco Federico^2^, Gianfranco Ferraccioli^1*^ and Elisa Gremese^1^

1. Division of Rheumatology - Fondazione Policlinico Universitario A. Gemelli IRCCS - Catholic University of the Sacred Heart - Rome, Italy
2. Institute of Pathology - Fondazione Policlinico Universitario A. Gemelli IRCCS - Catholic University of the Sacred Heart – Rome, Italy

***Corresponding author:**

Gianfranco Ferraccioli

Division of Rheumatology

Fondazione Policlinico Universitario A. Gemelli IRCCS

Catholic University of the Sacred Heart

Via Giuseppe Moscati, 31, 00168, Rome, Italy.

Email address: [gianfranco.ferraccioli@unicatt.it](mailto:gf1990@gmail.com)

**Supplementary Table 1.** Inter-rater agreement coefficients for CD68, CD20, CD3, CD21, CD31 IHC scores.

|  | **Inter-rater coefficient*** |
| --- | --- |
| **Lining CD68 IHC score** | R=0.52; p<0.001 |
| **Sublining CD68 IHC score** | R=0.36; p=0.02 |
| **Lining CD21 IHC score** | R=0.89; p<0.001 |
| **Sublining CD21 IHC score** | R=0.84; p<0.001 |
| **Lining CD20 IHC score** | R=0.53; p<0.001 |
| **Sublining CD20 IHC score** | R=0.70; p<0.001 |
| **Lining CD3 IHC score** | R=0.54; p<0.001 |
| **Sublining CD3 IHC score** | R=0.68; p<0.001 |
| **CD31^+^ vessels count** | R=0.67; p<0.001 |

**IHC**: Immunohistochemistry. *Pearson Correlation coefficient.
