# Supplementary material for: Synovial Predictors of Differentiation to Definite Arthritis in Patients With Seronegative Undifferentiated Peripheral Inflammatory Arthritis: microRNA Signature, Histological, and Ultrasound Features
Source: Front Med (Lausanne). 2018 Jul 3;5:186. doi: 10.3389/fmed.2018.00186 (PMC6037719; doi:10.3389/fmed.2018.00186)
Supplement: Supplementary file 2 [file Table_2.doc]

Supplementary Material

**Synovial predictors of differentiation to definite arthritis in patients with seronegative undifferentiated peripheral inflammatory arthritis: microRNA signature, histological and ultrasound features.**

Stefano Alivernini^1^, Barbara Tolusso^1^, Luca Petricca^1^, Laura Bui^2^, Clara Di Mario^1^, Maria Rita Gigante^1^, Gabriele Di Sante^1^, Roberta Benvenuto^2^, Anna Laura Fedele^1^, Francesco Federico^2^, Gianfranco Ferraccioli^1*^ and Elisa Gremese^1^

1. Division of Rheumatology - Fondazione Policlinico Universitario A. Gemelli IRCCS - Catholic University of the Sacred Heart - Rome, Italy
2. Institute of Pathology - Fondazione Policlinico Universitario A. Gemelli IRCCS - Catholic University of the Sacred Heart - Rome, Italy

***Corresponding author:**

Gianfranco Ferraccioli

Division of Rheumatology

Fondazione Policlinico Universitario A. Gemelli IRCCS

Catholic University of the Sacred Heart

Via Giuseppe Moscati, 31, 00168, Rome, Italy.

Email address: [gianfranco.ferraccioli@unicatt.it](mailto:gf1990@gmail.com)

**Supplementary Table 2. Demographic, clinical and inflammatory characteristics of enrolled UPIA patients whose synovial fluid was available at study entry.**

|  | **WHOLE**  **UPIA COHORT**  **(N=16)** | **DIFFERENTIATION** | | ***p*** |
| --- | --- | --- | --- | --- |
|  |  | **NO**  **(N=11)** | **YES**  **(N=5)** |  |
| **Gender, female n(%)** | 8 (50.0) | 5 (45.5) | 3 (60.0) | *0.58* |
| **Age, years (mean ± SD)** | 50.50 **±** 15.37 | 52.36 **±** 14.87 | 46.40 **±** 17.40 | *0.43* |
| **Sympthoms duration, months (mean ± SD)** | 19.25 **±** 16.63 | 18.91 **±** 14.54 | 20.00 **±** 13.94 | *0.65* |
| **ESR, mm/1^st^ hour (mean ± SD)** | 28.00 **±** 18.98 | 25.40 **±** 18.53 | 33.20 **±** 20.92 | *0.33* |
| **CRP, mg/L (mean ± SD)** | 12.27 **±** 16.27 | 10.82 **±** 12.37 | 15.18 **±** 23.77 | *0.90* |
| **Swollen Joint count, (mean ± SD)** | 2.25 **±** 1.57 | 1.82 **±** 1.77 | 3.19 **±** 2.11 | *0.31* |
| **NSAIDs usage, n(%)** | 11 (68.8) | 6 (54.5) | 5 (100.0) | *0.07* |
| **GC usage, n(%)** | 0 (0.0) | 0 (0.0) | 0 (0.0) | *1.00* |
| **Smoking habit, n(%)** | 6 (37.5) | 4 (36.4) | 2 (40.0) | *0.89* |
|  |  |  |  |  |
| **Diagnosis after differentiation** |  |  |  |  |
| **UPIA ⇒ RA** | - | - | 1 (20.0) | ***-*** |
| **UPIA ⇒ PsA** | - | - | 2 (40.0) | ***-*** |
| **UPIA ⇒ SpA** | - | - | 2 (40.0) | ***-*** |

**UPIA**: Undifferentiated Peripheral Inflammatory Arthritis; **ESR**: Erytrocyte Sedimentation Rate; **CRP**: C-Reactive Protein; **NSAIDs**: Non Steroideal Anti-Inflammatory Drugs; **GC**: corticosteroids; **RA**: Rheumatoid Arthritis; **PsA**: Psoriatic Arthritis; **SpA**: Spondyloarthritis; **SD:** Standard Deviation. ***p value***: Mann-Whitney test comparing UPIA patients who reached a defined clinical diagnosis during the follow-up (differentiation) vs patients who remained as UPIA after 1 year follow-up (no differentiation).
