# Supplementary material for: Synovial Predictors of Differentiation to Definite Arthritis in Patients With Seronegative Undifferentiated Peripheral Inflammatory Arthritis: microRNA Signature, Histological, and Ultrasound Features
Source: Front Med (Lausanne). 2018 Jul 3;5:186. doi: 10.3389/fmed.2018.00186 (PMC6037719; doi:10.3389/fmed.2018.00186)
Supplement: Supplementary file 3 [file Table_3.doc]

Supplementary Material

**Synovial predictors of differentiation to definite arthritis in patients with seronegative undifferentiated peripheral inflammatory arthritis: microRNA signature, histological and ultrasound features.**

Stefano Alivernini^1^, Barbara Tolusso^1^, Luca Petricca^1^, Laura Bui^2^, Clara Di Mario^1^, Maria Rita Gigante^1^, Gabriele Di Sante^1^, Roberta Benvenuto^2^, Anna Laura Fedele^1^, Francesco Federico^2^, Gianfranco Ferraccioli^1*^ and Elisa Gremese^1^

1. Division of Rheumatology - Fondazione Policlinico Universitario A. Gemelli IRCCS - Catholic University of the Sacred Heart - Rome, Italy
2. Institute of Pathology - Fondazione Policlinico Universitario A. Gemelli IRCCS - Catholic University of the Sacred Heart - Rome, Italy

***Corresponding author:**

Gianfranco Ferraccioli

Division of Rheumatology

Fondazione Policlinico Universitario A. Gemelli IRCCS

Catholic University of the Sacred Heart

Via Giuseppe Moscati, 31, 00168, Rome, Italy.

Email address: [gianfranco.ferraccioli@unicatt.it](mailto:gf1990@gmail.com)

**Supplementary Table 3.** ROC curves for IL-6, CD68, CD3 and CD31 cut-off values and GS and PDUS cut-off values associated with differentiation in UPIA patients.

|  | **AUC (95%CI)** | **P value** | **Cut-off value** | **Sensitivity** | **Specificity** |
| --- | --- | --- | --- | --- | --- |
| **CD31^+^ vessels number** | 0.981 (0.94-1.00) | *0.001* | 24.3 | 83.3 | 97.2 |
| **CD68^+^ lining score** | 0.847 (0.70-0.99) | *0.01* | 2.16 | 50.0 | 94.4 |
| **CD68^+^ sublining score** | 0.764 (0.55-0.98) | *0.04* | 1.16 | 83.3 | 69.4 |
| **CD3^+^sublining score** | 0.880 (0.77-0.99) | *0.003* | 1.16 | 83.3 | 83.3 |
| **IL-6 PB levels** | 0.902 (0.78-1.0) | *0.01* | 4.75 | 100 | 78.3 |
| **Gray scale score** | 0.813 (0.32-1.0) | *0.02* | 1.5 | 83.3 | 75.0 |
| **Power Doppler score** | 0.806 (0.571-1.0) | *0.02* | 1.5 | 66.7 | 94.4 |

**AUC**: Area Under the Curve; **CD**: cluster differentiation. **GS**: Gray Scale; **PD**: Power Doppler; **US**: Ultrasound. **PB**: Peripheral Blood; **IL**: interleukin.
