# Supplementary material for: Synovial Predictors of Differentiation to Definite Arthritis in Patients With Seronegative Undifferentiated Peripheral Inflammatory Arthritis: microRNA Signature, Histological, and Ultrasound Features
Source: Front Med (Lausanne). 2018 Jul 3;5:186. doi: 10.3389/fmed.2018.00186 (PMC6037719; doi:10.3389/fmed.2018.00186)
Supplement: Supplementary file 4 [file Image_1.PDF]

Supplementary Figure 1. Correlation plots between US parameters (GSUS and PDUS) and IHC scores of synovial tissue inflammatory cells.

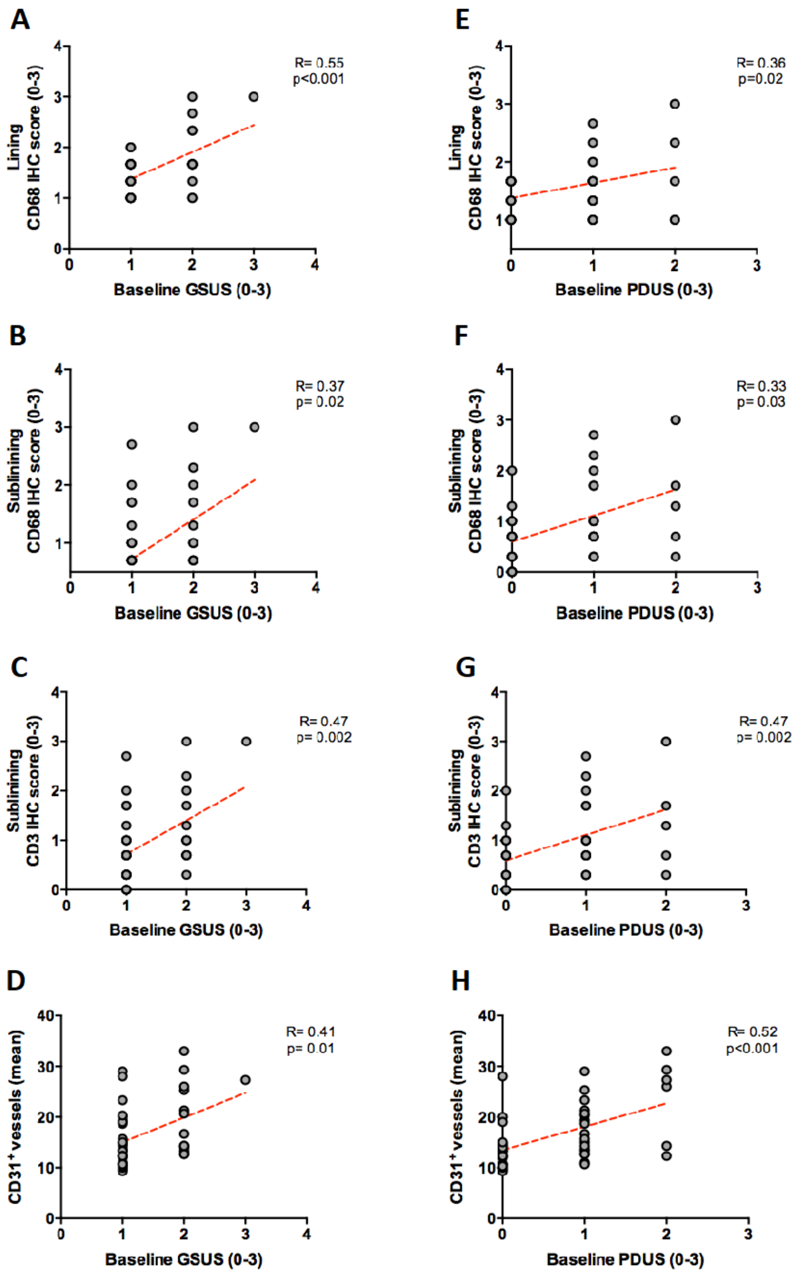

Supplementary Figure 1 legend: Correlations between GSUS score and IHC scores of **(A)** lining $CD68^{+}$ cells, **(B)** sublining $CD68^{+}$ cells, **(C)** sublining $CD3^{+}$ cells and **(D)** mean number of $CD31^{+}$ synovial vessels; Correlations between PDUS score and IHC scores of **(E)** lining $CD68^{+}$ cells, **(F)** sublining $CD68^{+}$ cells, **(G)** sublining $CD3^{+}$ cells and **(H)** mean number of $CD31^{+}$ synovial vessels; **GSUS**: Gray-Scale Ultrasound; **PDUS**: Power-Doppler Ultrasound.
