# Supplementary material for: Synovial Predictors of Differentiation to Definite Arthritis in Patients With Seronegative Undifferentiated Peripheral Inflammatory Arthritis: microRNA Signature, Histological, and Ultrasound Features
Source: Front Med (Lausanne). 2018 Jul 3;5:186. doi: 10.3389/fmed.2018.00186 (PMC6037719; doi:10.3389/fmed.2018.00186)
Supplement: Supplementary file 5 [file Image_2.PDF]

Supplementary Figure 2. Unsupervised hierarchical clustering of microRNAs in UPIA patients based on their differentiation during the follow-up.

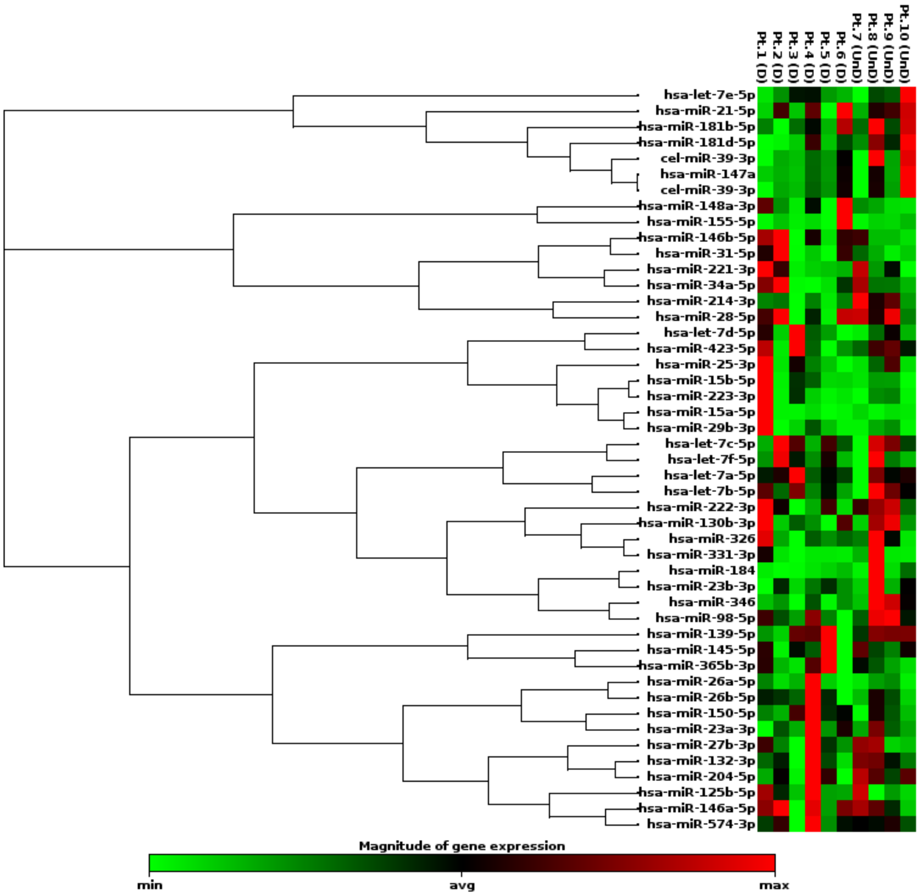

Supplementary Figure 2 legend: Unsupervised hierarchical clustering generated using fold change expression values of microRNAs analysed in UPIA patients who reached a definite diagnosis during the follow-up (D) versus UPIA patients who remained as UPIA (UnD); Color scheme: green: decreased expression; Red: Increased expression; Black: Unchanged.
